# Adrenal Insufficiency Secondary to Monkeypox Infection: A Case Report

**DOI:** 10.7759/cureus.39372

**Published:** 2023-05-23

**Authors:** Wael A Mohammed, Mohamed A Zaki

**Affiliations:** 1 Cardiology, London North West Healthcare NHS Trust, London, GBR; 2 Medicine, Ysbyty Glan Clwyd Hospital, Betsi Cadwaladr University Health Board, Rhyl, GBR

**Keywords:** monkeypox, postural orthostatic hypotension, monkey pox virus rash, disseminated disease, adrenal disease, monkey pox virus

## Abstract

Our case report confirms adrenal insufficiency following three weeks of monkeypox infection in an immunocompetent patient. As there is no specific treatment for monkeypox, steroid replacement therapy remains the cornerstone of managing adrenal insufficiency. By highlighting the importance of early identification and tracing in our case report, we contribute to the growing body of knowledge on monkeypox and emphasize the importance of identifying adrenal insufficiency and other complications like meningoencephalitis, prompting a significant early intervention that can help manage these complications effectively, potentially improving patient outcomes. In addition, understanding disease progression by tracing monkeypox cases provides valuable information on the disease's natural history, including the timeline and sequence of symptoms, associated complications, and outcomes, to avoid any small chance related to our lack of validated knowledge or incorrect assessment of the disease or the associated complications between an exposure and an effect in the target organs.

## Introduction

Monkeypox is an orthopoxvirus, which is a DNA virus related to the group that causes smallpox. It was first detected in mankind in 1970 in Africa, more specifically in the Congo (previously known as Zaire) [1-6]. The World Health Organization (WHO) announced that the monkeypox disease is raising public health concerns in June 2022 [3]. The monkeypox incubation period usually takes up to three weeks. Patients are infectious from the time fever and rashes start; they often start on the face and then spread to other parts of the body [7]. Lesions progress from macular to papular to vesicular to pustular stages until their lesions have crusted and scabs have fallen off to reveal healthy skin [7].

Adrenal insufficiency is a gland disorder that occurs when the virus causes the destruction of the adrenal cortices, leading to deficiencies of certain hormones like glucocorticoids (cortisol), mineralocorticoids (aldosterone), and steroids. This can cause symptoms such as fatigue, weakness, weight loss, low blood pressure, and electrolyte imbalances. However, it's important to note that not all people with monkeypox infection develop adrenal insufficiency, and it is not a common complication of the disease.

## Case presentation

**Figure 1:**
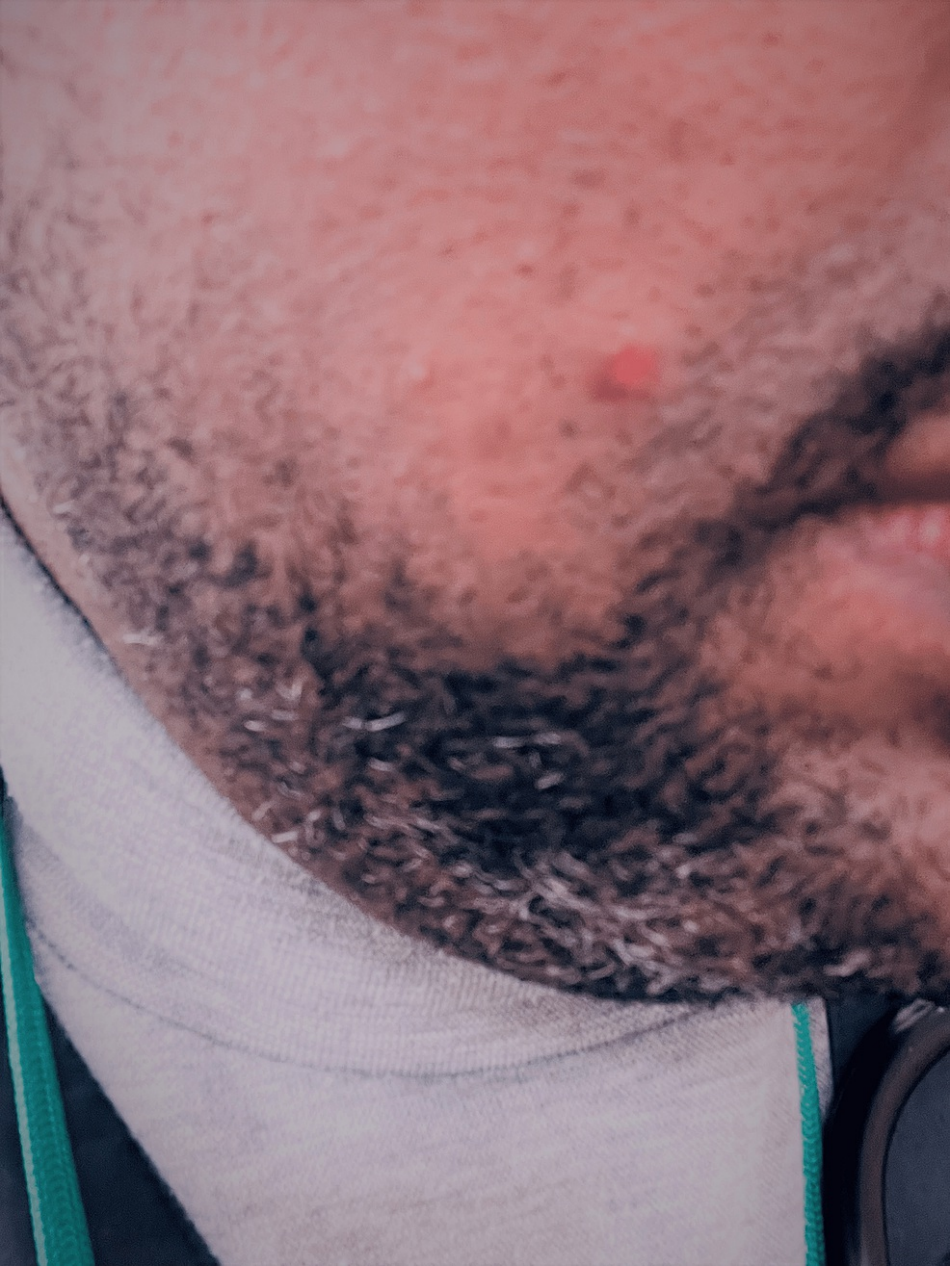
Monkeypox skin lesions on the patient's face

**Figure 2:**
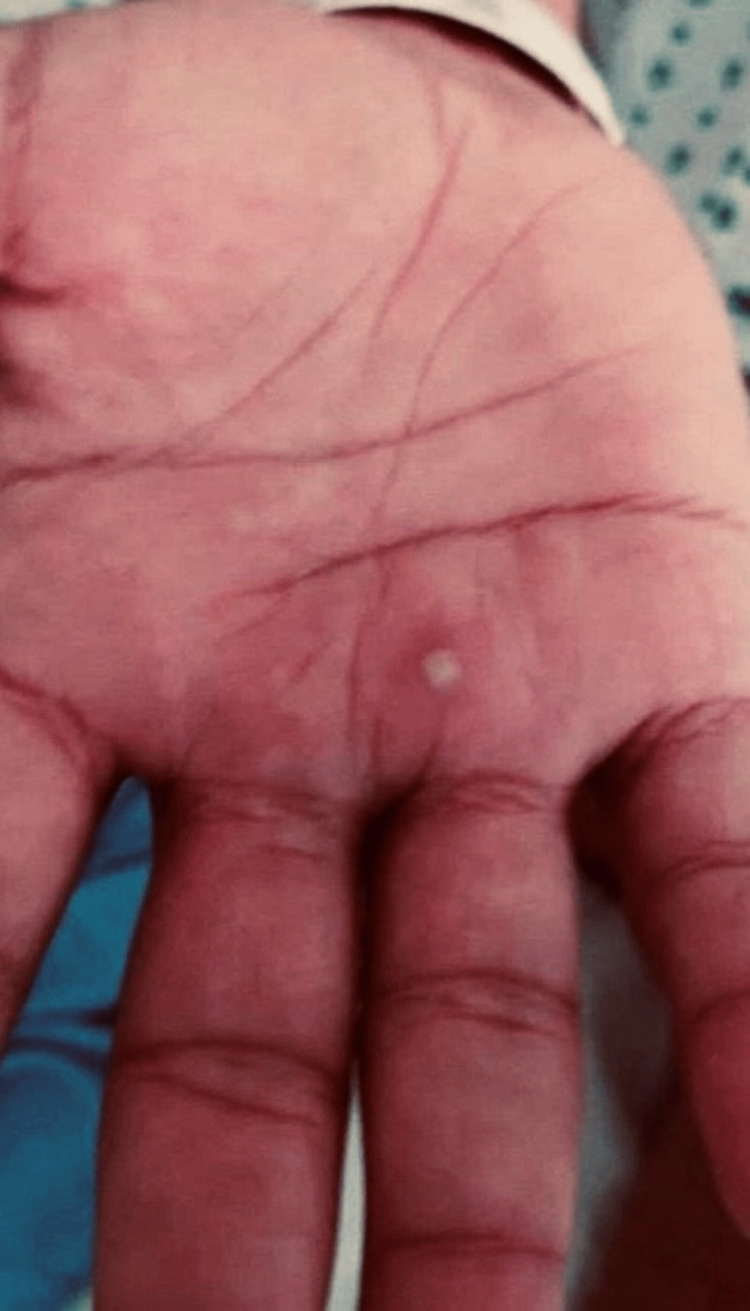
Monkeypox skin lesions on the patient's palm

A 21-year-old male flight attendant arrived from Spain at the end of June 2022. He had a history of depression, bronchial asthma, and atopic dermatitis, for which he had been receiving amitriptyline 10mg and sertraline 100mg orally and dupilumab 300mg subcutaneously every two weeks for more than two years, but he never required short- or long-term steroid therapy. He presented initially on July 19, 2022, with a four-day history of very few blistering skin lesions on his face and palms of the hands (Figures 1-2). His monkeypox polymerase chain reaction (PCR) test came back positive (Table 1).

**Table 1:** Laboratory investigation PCR: polymerase chain reaction; Hb: hemoglobin; WBC: white blood cell; PLT: platelets; CRP: C-reactive protein

Tests	Results	Reference value
Monkeypox PCR DNA detected 1	Positive	Negative
Herpes simplex type 1 PCR	Negative	Negative
Herpes simplex type 2 PCR	Negative	Negative
Varicella zoster PCR	Negative	Negative
Skin enterovirus PCR, glandular fever screen	Negative	Negative
Swab for *Haemophilus ducreyi* (HD)	Negative	Negative
Lymphogranuloma venereum (LGV) antibodies	Negative	Negative
Treponema pallidum (TP) antibodies	Negative	Negative
WBC (X10^9/l)	10	(4.5 - 11)
Hb (g/dL)	14.1	(13.5 - 17.5)
PLT (X10^3/ul)	279	(150-450)
Esinophilsx10^9/L	0.6	(0-0.5)
CRP (mg/L)	7	(0-5)

Thus, he was advised to stay in home isolation until August 7 and to avoid handling clothes, sheets, or other materials that have been in contact with him with other people. At that time, his symptoms included a sore throat and fatigue. On August 1, he visited the accident and emergency (A&E) department following a severe headache, photophobia, and eye conjunctivitis. He was isolated and admitted for further investigations and treatment. Clinical examination demonstrated neck stiffness, and he was isolated for suspected meningoencephalitis, but the patient refused lumber puncture and cerebrospinal fluid (CSF) analysis.

A computerized tomography scan of the head was normal, and he was started on ceftriaxone and acyclovir. Subsequently, the patient’s condition improved, along with inflammatory markers. His full virology screen panels were unremarkable (Table 1).

He was seen and treated by a multidisciplinary team, including an infectious disease consultant, an ophthalmologist, and an internist. At the end of the antimicrobial regimen, dizziness and malaise were reported, and a significant postural drop in blood pressure was recorded. Based on that, a morning cortisol level was checked and revealed a low level of 64.89 nmol/L (normal range: 140-690 nmol/L). An urgent endocrinology consultation was obtained, and the synacthen test was performed the next day and showed a positive response (Table 2).

**Table 2:** Laboratory investigations PCR: polymerase chain reaction, CT/CTA: Computed Tomography Angiography, ACTH: adrenocorticotropic hormone, ALT: alanine transaminase, AST: aspartate aminotransferase, BUN: blood urea nitrogen, Cr: creatinine, Na: serum sodium, K: serum potassium, TSH:  thyroid stimulating hormone, FSH: follicle-stimulating hormone, LH: luteinizing hormone

Tests	Results	Reference value
Meningococcal PCR	DNA not detected	Undetected
Pneumococcal PCR		Undetected
Coronavirus SARS CoV 2 PCR	DNA not detected	Undetected
CT/CTA Brain	Normal appearances of anterior cerebral arteries. No vascular or filling defects noted	
Corisol at 08:00am (nmol/L)	18	(83 - 359)
Cortisol at 08:30mins (nmol/L)	499	≥450 nmol/L Adequate response to 250ug synacthen
ACTH (ng/L)	3.9	(1 - 50)
BUN (mg/dL)	18	(6 - 25)
Cr (mg/dL)	0.9	(0.7 - 1.3)
Na (mEq/L)	137	(135 - 145)
K (mEq/L)	4.8	(3.5 - 5)
ALT (IU/L)	24	(5 - 45)
AST (IU/L)	33	(5 - 45)
TSH (mU/L)	2.2	(0.38 - 5.33)
FSH (IU/L)	4.8	(1.3 - 19.3)
LH (IU/L)	2.4	(1.2 - 8.6)
Prolactin (mIU/L)	98	(less < 325)

**Figure 3:**
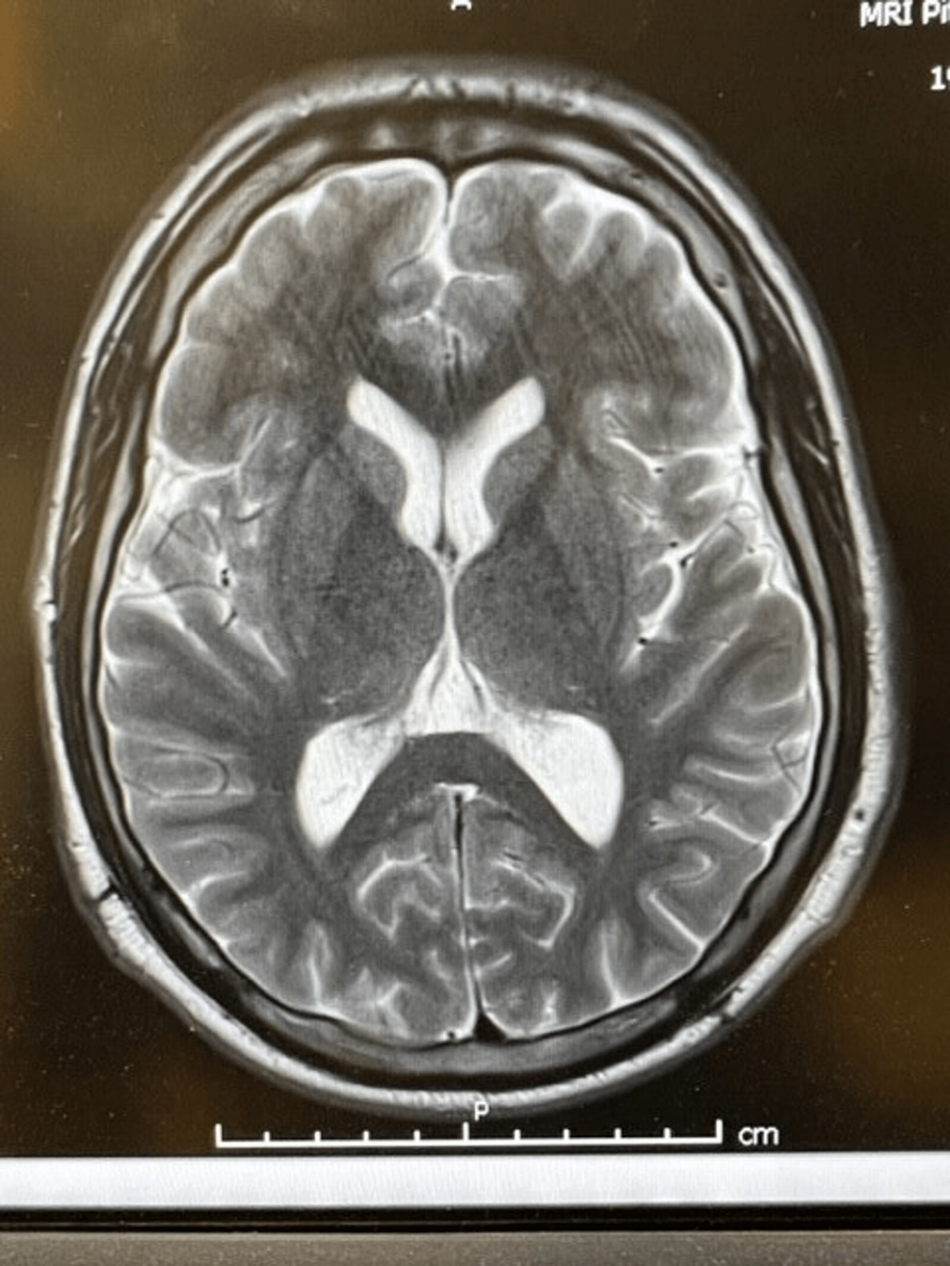
Brain MRI

Furthermore, an MRI of the brain was unremarkable (Figure 3).

**Figure 4:**
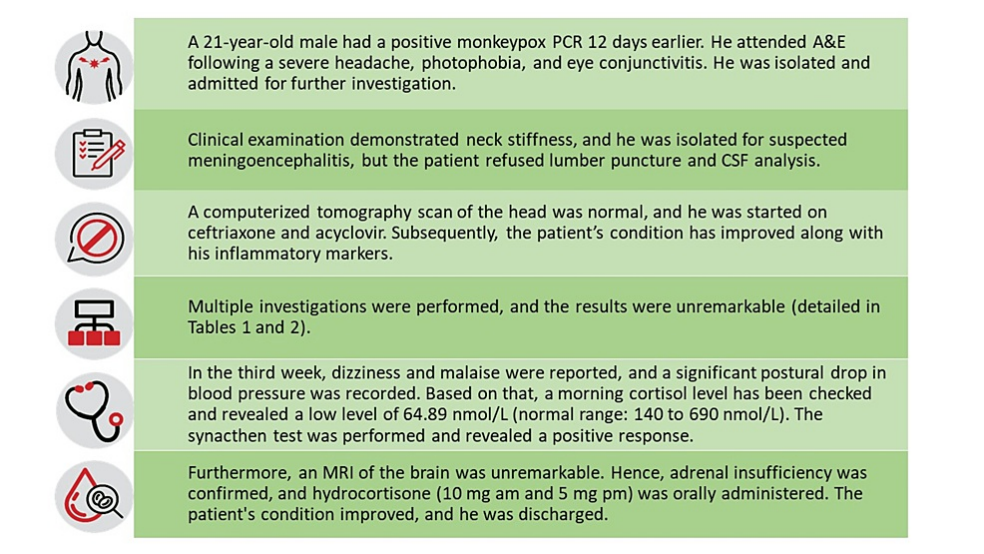
Case summary

Hence, adrenal insufficiency was confirmed, and hydrocortisone, 10 mg in the morning and 5 mg in the evening (physiological doses), was orally administered. The patient improved and reported no symptoms; thereafter, he was discharged home on August 19, 2022. The case summary is presented in Figure 4.

## Discussion

The monkeypox disease has typical clinical features; however, complications vary. It usually starts with a fever, progressing to the development of multiple skin lesions that progress from macular to papular to vesicular to pustular stages and are associated with lymphadenopathy. It often starts on the face and then spreads to other parts of the body [7]. Complications include encephalitis, pneumonitis, conjunctivitis, keratitis, and secondary bacterial infections. Two types of serious complications were recently reported: one case of epiglottitis and two cases of myocarditis. No mortality was reported [5].

Adrenal insufficiency is a potential complication of monkeypox infection. The adrenal glands, which produce hormones essential for various bodily functions, can be affected by the virus. For these cases, a random cortisol test would be a helpful strategy, and the synacthen test is the gold standard if this complication is suspected. Steroid therapy can be safely introduced to these patients.

Until now, there have been no specific antiviral treatments approved for monkeypox. It's also important to note that treatment and prevention strategies for monkeypox should be tailored to each individual's specific circumstances, including their medical history and any underlying health conditions. Despite the fact that monkeypox can be serious, most people completely recover. Currently, two medications, such as ST-246 and brincidofovir, together with vaccines like JYNNEOS and ACAM2000, have been approved by the United States Food and Drug Administration (FDA) for certain high-risk populations. Although it gives partial protection, it is not routinely advised for the general population. The absence of widely available treatment or prophylaxis underscores the importance of rapid case identification and isolation. Monkeypox treatment is mainly supportive, as most cases are self-limiting. However, such complications as adrenal insufficiency should always be kept in mind. The exact timing of the identification of monkeypox complications before and after skin lesions remains uncertain. In the case of any suspicion or change in the patient’s condition (presence of new symptoms), a multidisciplinary approach is quite helpful. In addition, frequent blood tests need to be performed, depending on the patient’s clinical situation.

There are a few reasons why monkeypox may have received less attention and funding compared to other infectious diseases. First of all, the prevalence is partly because monkeypox is considered a rare disease and primarily affects individuals in Central and West Africa, where it has traditionally been endemic. Secondly, global health is a priority due to its less significant impact on public health and low mortality rates. Lastly, the effective eradication of smallpox has shifted the focus of public health efforts away from poxviruses like monkeypox. However, the recent outbreaks of monkeypox in various regions of the world have drawn attention to the need for more research into the virus. The outbreaks have demonstrated that the virus is not confined to endemic regions and can pose a potential threat to global health security. The increase in reported cases, including imported cases in non-endemic regions, has sparked interest and raised concerns about its potential for international spread. This has led to a renewed focus on understanding the virus, improving diagnostics, and developing strategies for prevention and control.

Increased funding and research into monkeypox could help improve our understanding of the virus, its transmission, and the development of effective vaccines and treatments. Additionally, it could help improve early detection and response to future outbreaks, which are crucial for containing the spread of the virus and preventing its global spread. Ultimately, the virus knows no borders, and the disease manifestation rarely becomes complex; therefore, we need to close knowledge gaps and enhance cooperation between nations to effectively manage the outbreaks of monkeypox and its complications.

## Conclusions

Monkeypox is a viral disease that can cause significant morbidity and mortality, and early recognition of the disease and its complications, such as adrenal insufficiency, is crucial. Our goal is to remind all the medical professionals who are dealing with suspected individuals with monkeypox that they should be aware of the signs and symptoms of the disease and its complications. and be prepared to manage these cases effectively. This information contributes to a better understanding of the disease and helps healthcare professionals refine diagnostic criteria, treatment guidelines, and surveillance strategies.

## References

[1] Ladnyj ID, Ziegler P, Kima E (1972). A human infection caused by monkeypox virus in Basankusu Territory, Democratic Republic of the Congo. Bull World Health Organ.

[2] Bunge EM, Hoet B, Chen L, Lienert F, Weidenthaler H, Baer LR, Steffen R (2022). The changing epidemiology of human monkeypox- a potential threat? A systematic review. PLoS Negl Trop Dis.

[3] (2022). Mpox (monkeypox). https://www.who.int/news-room/fact-sheets/detail/monkeypox.

[4] Orviz E, Negredo A, Ayerdi O (2022). Monkeypox outbreak in Madrid (Spain): clinical and virological aspects. J Infect.

[5] Thornhill JP, Barkati S, Walmsley S (2022). Monkeypox virus infection in humans across 16 countries - April-June 2022. N Engl J Med.

[6] Vivancos R, Anderson C, Blomquist P (2022). Community transmission of monkeypox in the United Kingdom, April to May 2022. Euro Surveill.

[7] Jayasinghe M, Caldera D, Prathiraja O (2022). Waking up to monkeypox in the midst of COVID-19. Cureus.

